# Data assimilation and agent-based modelling: towards the incorporation of categorical agent parameters

**DOI:** 10.12688/openreseurope.14144.2

**Published:** 2022-07-20

**Authors:** Patricia Ternes, Jonathan A Ward, Alison Heppenstall, Vijay Kumar, Le-Minh Kieu, Nick Malleson

**Affiliations:** 1 Leeds Institute for Data Analytics, University of Leeds, Leeds, UK; 2 School of Geography, University of Leeds, Leeds, UK; 3 School of Mathematics, University of Leeds, Leeds, UK; 4 Alan Turing Institute, London, UK; 5 School of Political and Social Sciences, University of Glasgow, Glasgow, UK; 6 MRC/CSO Social and Public Health Sciences Unit, University of Glasgow, Glasgow, UK; 7 Department of Civil and Environmental Engineering, University of Auckland, Auckland, New Zealand

**Keywords:** Agent-Based Modelling, Crowd Simulation, Data Assimilation, Particle Filter

## Abstract

This paper explores the use of a particle filter—a data assimilation method—to incorporate real-time data into an agent-based model. We apply the method to a simulation of real pedestrians moving through the concourse of Grand Central Terminal in New York City (USA).  The results show that the particle filter does not perform well due to (i) the unpredictable behaviour of some pedestrians and (ii) because the filter does not optimise the categorical agent parameters that are characteristic of this type of model. This problem only arises because the experiments use real-world pedestrian movement data, rather than simulated, hypothetical data, as is more common. We point to a potential solution that involves resampling some of the variables in a particle, such as the locations of the agents in space, but keeps other variables such as the agents’ choice of destination. This research illustrates the importance of including real-world data and provides a proof of concept for the application of an improved particle filter to an agent-based model.  The obstacles and solutions discussed have important implications for future work that is focused on building large-scale real-time agent-based models.

## Plain language summary

The use of computer models to simulate the movement of a crowd of people through a busy environment, like a train station or city centre, is a useful way to manage busy spaces. The technique of *agent-based modelling* is often used to simulate crowds. This method simulates the actions of all of the people that might be in a crowd to try to predict how the whole crowd will behave. One problem with agent-based modelling is that it is very difficult to adapt a simulation in response to new data. This means that it is not possible to use the technique to simulate crowds *in real time*.

This paper experiments with a method that can be used to optimise an agent-based crowd simulation in real time called the particle filter. We show that the particle filter is not able to model the behaviour of virtual people (called ‘agents’) in some circumstances, particularly when the agents have characteristics that cannot be represented on a numeric scale, such as their chosen destination. We propose a potential solution and run experiments to test its reliability. Although there is still a lot of work to do before the method can be used in practice, this paper makes an important first step towards a better understanding of how to use a particle filter to create real-time crowd simulations.

## Introduction

Agent-based modelling (ABM) is an interdisciplinary field that has emerged from the study of nonlinear, complex adaptive systems and computational modelling. A defining characteristic of an agent-based model is the simulation of discrete, heterogeneous and autonomous *agents* ^1^. This is particularly relevant for the study of short-term human movements, such as pedestrian dynamics, where higher-level features such as crowding emerge from interactions and behaviour of many individual people.

One of the biggest difficulties in modelling pedestrian behaviour in *real time* is that the inherent complexity of the system causes a model to quickly diverge from reality. This uncertainty—i.e. the imprecision in modelling outputs ^2^—can be reduced through robust parameter calibration. A considerable body of work has gone in to calibrating agent-based models in general (e.g. 3) and pedestrian models in particular ^4^. However, even perfectly-calibrated models of complex systems will diverge over time ^5^ as their underlying systems evolve. In agent-based models these problems are intensified since each agent has a set of parameters (the parameters are not global) and, generally, the available data do not have all the necessary spatio-temporal information to cover both individual actions and global behaviour. Despite the progress that has been made in the static calibration of ABMs ^3, 6^, little progress has been made towards methods that can control the uncertainty in models *during run time*. This makes it hard to predict systems that rapidly evolve, such as pedestrian systems, in real-time using agent-based models.

Fortunately, methods developed in other fields potentially offer solutions to the problems of real-time model updating in response to new observations. The technique of ‘data assimilation’ ^7^ (DA), which has been well studied in highly computational fields such as meteorology ^7^ and the earth sciences more broadly ^8^, is a primary candidate. DA is founded on the premise that observations of a system are relatively certain but sparse (sensors only generate data at discrete points), and modelled estimates of a system are detailed but uncertain. By combining observations and model estimates in real time "all the available information" ^9^ can be used to estimate the current *true* state of the underlying system. The approach is distinct from traditional ‘one-shot’ optimisation approaches; DA algorithms use real-time data to constrain the model’s *continued evolution* against observations of the real world ^10^. In short, whereas calibration methods only optimise model parameters, DA techniques optimise the parameters *and the model state itself*.

In recent years, efforts have been made towards the use of DA techniques with agent-based modelling ^10– 17^. The results are promising, although limited by the controlled nature of the experiments. Most experiments make use of the identical twin experimental framework to obtain the ground truth state to be used as the real world ‘observation’ during DA steps ^11, 12, 14– 17^. This constraint implies that pseudo-real observations will never exhibit a behaviour that cannot be fully described by the model; ignoring the underlying complexity and uncertainty inherent in many social systems, including pedestrian behaviour. The identical twin approach also allows for controlling the number of entities present in the observations, and as a consequence, the number of simulated agents is normally kept in the order of units ^11^ or a few dozen ^12, 14– 17^. Another common constraint is related to the model environment, including the use of one-dimensional environments ^12, 15^ and comprehensive simplifications of real environments ^14, 16^. Usually model environment simplifications are combined with identical twin experiments, which, again, implies a great deal of control over the observations and of the agents’ behaviours. Finally, although categorical parameters are common in ABMs, the majority of the agent-based models used to test DA do not include this type of parameter in their formulation, and the main reason is related to the fact that DA methods have been developed for equation based models ^10, 17^.

This paper extends the state-of-the-art by experimenting with DA and agent-based models under more realistic assumptions. For this, we use an ABM that simulates simple pedestrian movements across a train station, the *StationSim* model, and a well-known DA technique, the particle filter (discussed below) ^18^. The model environment is designed to represent a real station – the Grand Central Terminal in New York City, USA – the observations used reflect real pedestrian behaviour and have been inferred from a CCTV camera at GCT, the model has both continuous and categorical parameters and hundreds of agents are considered.

There are a range of DA techniques that could be used to assimilate data from Grand Central Terminal into the *StationSim* model. Variations of the Kalman Filter are popular ^11, 16, 17, 19^, but may not be entirely appropriate for application to ABM because they rely on Gaussian assumptions that do not hold in highly non-linear ABMs. Instead, we chose the particle filter (PF) ^18^ (another well-known DA technique) because they have been shown to work well with non-linear systems and, because they are decoupled from the underlying model, should work well in situations where state corrections in the filter are difficult ^19^. Although PFs have been in development for some decades ^20^, they have only been applied to ABMs a handful of times ^14, 21^, and are still in their infancy with respect to their use in high-dimensional applications.

Interestingly, the vanilla PF implementation actually *increases* the error in the simulated agent positions because the complexity of some of the pedestrians’ behaviour means that the filter inadvertently filters out some important particles initially. Hence, before concluding, we trial an adapted approach that attempts to prevent some useful parameter values from being inadvertently filtered out. This is an important result with respect to future work.

The paper is structured as follows: first all necessary methods and simulation details are defined, then the experiments and results are presented, and, finally, the results are discussed and conclusions are drawn. The software codes that underpin the work discussed here and instructions for operating the codes are available in full from the project code repository ^22^.

## Methods

### StationSim

**Figure 1.**
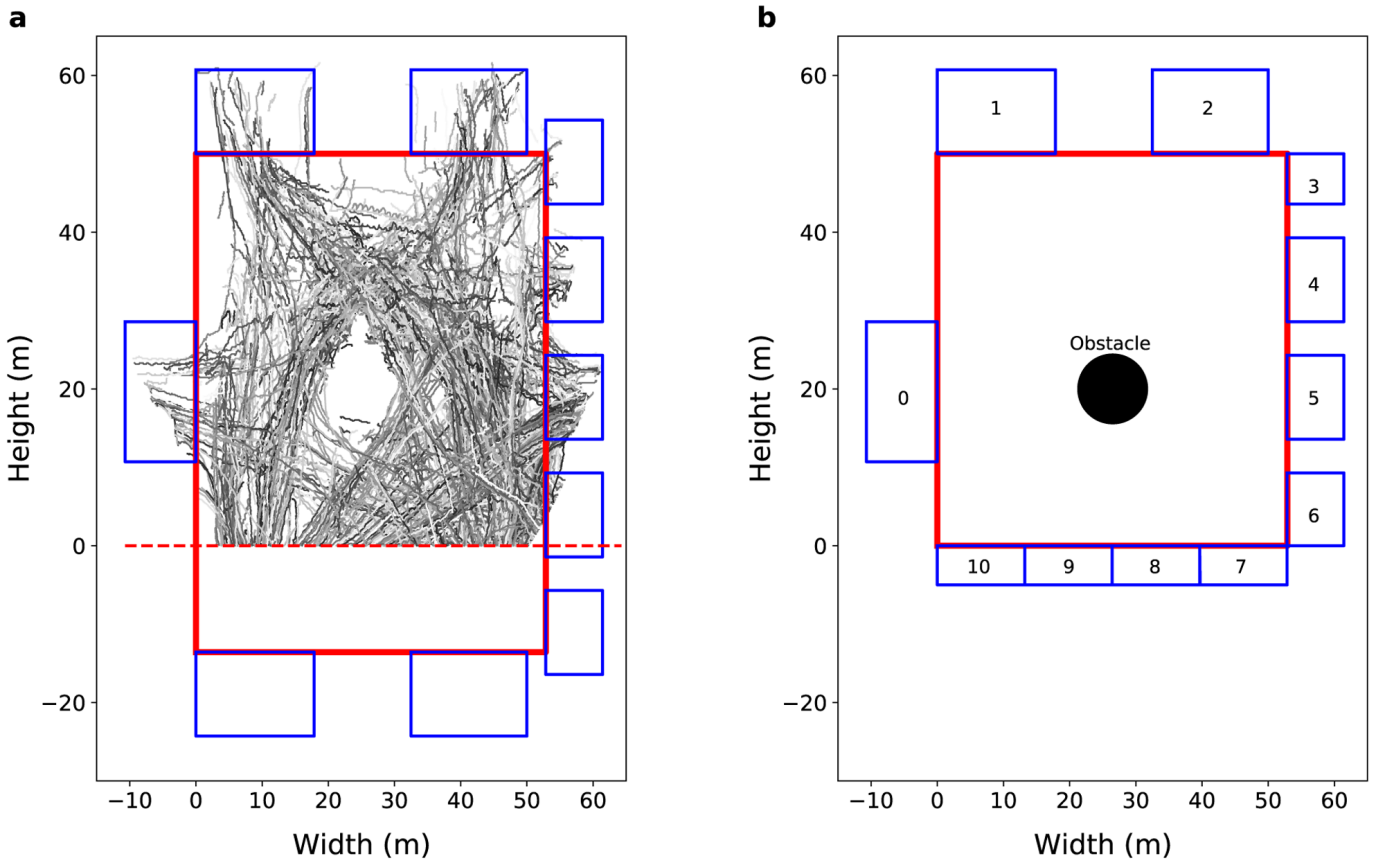
General environment view. ( **a**) View from the Grand Central Terminal (GCT). The grayscale lines represent real pedestrian trajectories. The solid red line represents the station walls, the blue rectangles represents the 10 gates of the GCT concourse, and the red dashed line separate the camera’s field of view from non-visible region. ( **b**) General view of the model environment. The solid red lines represent the station walls, the blue rectangles represent the gates, and the black circle represents an obstacle in the centre of the concourse.

The model environment is rectangular with entry and exit gates on the environment boundaries (see Figure 1). Each agent represents a pedestrian that enters the system through one of the gates ( *entrance gate*) in a certain simulation step ( *activation time*) and tries to walk towards an assigned destination ( *exit gate*) with constant speed ( *desired speed*). The behaviour of each agent is determined through three rules ^17^:

1. The agent will try to walk in a straight line (ideal path) towards the destination;
2. If the ideal path is blocked, the agent will try to take a side step to avoid collisions with other agents or the environment;
3. If none of the above options are possible, the agent will stand still.

These rules are implemented following ^17^. In this implementation, each agent is represented by a disc of fixed radius *σ* and the collisions are identified through hard disc dynamics equations ^23^. In StationSim, collisions are defined as an overlap between two agents (or between an agent and the environment) within the next simulation step *δt*.

Mathematically, the time until overlapping between two agents is


$$\;\text{Δ}t=\frac{-\text{Δ}\vec{v}\cdot \text{Δ}\vec{r}-\sqrt{d}}{\text{Δ}\vec{v}\cdot \text{Δ}\vec{v}},\;(1)$$

where $\text{Δ}\vec{r}$ is the difference between the agents positions, $\text{Δ}\vec{r}$ is the difference between the agents velocities, and $d={\left(\text{Δ}\vec{v}\cdot \text{Δ}\vec{r}\right)}^{2}-(\text{Δ}\vec{v}\cdot \text{Δ}\vec{v})(\text{Δ}\vec{r}\cdot \text{Δ}\vec{r}-{\left(\sum_{i}\sigma_{i}\right)}^{2})$ . Note that if *d* < 0 the agents never collide with each other. The time until overlapping between an agent and a vertical wall is


$$\;\text{Δ}t=\frac{w_{x}\pm \sigma -x}{v_{x}},\;(2)$$

where *w _x_* is the vertical wall *x*-position, *x* is the agent horizontal position, and *v _x_* is the agent horizontal velocity. The *±* sign in Equation 2 is related to the agent direction of movement: if the agent is moving towards the left wall, a positive sign is used, and if the agent is moving towards the right wall, a negative sign is used. Note that if the agent does not have a horizontal movement, then the agent never collides with a vertical wall. The overlap time between an agent and a horizontal wall can be obtained in an analogous way.

If no collision is identified, i.e. the time until overlapping with another agent or environment, *∆t*, is greater than *δt*, then the agent will walk in the ideal path (rule 1). Otherwise, the agent will try to move sideways to avoid the collision. The direction (perpendicular to the left or right) is determined by a random binary choice, and the size of the movement is determined randomly from a normal distribution centred on the agent radius. The agent will only step sideways if the new location is empty and within the defined environment (rule 2), otherwise the agent will stand and wait until it is possible to move (rule 3).

Although the behaviour of each agent is determined through three simple rules, the interaction between the agents and the random choices that arises from these interactions can lead to different scenarios, including the formation of crowds. Furthermore, the behaviour of each agent depends on two initial attributes ( *entrance gate* and *activation time*), a continuous parameter ( *desired speed*), a categorical parameter ( *exit gate*), and a dynamic position. Finally, the state of an agent *i* at time *t* is defined as


$$\;s_{t}^{i}=\{{\vec{r}}_{t}^{i},\;v_{i},\;{\hat{g}}_{t}^{i}\},\;(3)$$

where ${\vec{r}}_{i,t}$ is the agent *i* position at time *t*, ${\hat{g}}_{i,t}$ is a normal vector that indicates the agent *i* direction of movement towards the *exit gate* at time *t*, and *v _i_* is the agent *i desired speed*. The simulation ends when all agents pass through their exit gate. Importantly, the ${\hat{g}}_{i,t}$ value is related to the *exit gate* that is a categorical parameter and, as we will discuss, presents difficulties for the traditional PF.

### Data & model parameterisation

This section outlines the real data used and the process of estimating suitable parameters for StationSim to allow the agent-based model to simulate the dynamics of the underlying system. GCT, a rail and subway hub in Manhattan, New York City (USA), has been chosen for two reasons. Firstly, it is a large, busy terminal, so presents a useful test of the ability for a DA algorithm to update the state of a crowd simulation in real time. Secondly, there is a readily available data set ^24^ that was created from CCTV footage and describes the pedestrians’ trajectories across the main concourse of the terminal, so we have detailed information about the underlying crowd on which to test the algorithm.

The original GCT video is 33 minutes and 20 seconds long at 25 fps with a resolution of 720 *×* 480. The video was first processed by 24 who determined the raw trajectories using a Kanade-Lucas-Tomasi (KLT) keypoint tracker ^25^. The raw trajectories were further processed by 17 to correct the camera distortions and to merge trajectories that had been inadvertently split when the video data was first processed. (‘trajectory reconstruction’). The trajectory reconstruction process needed to be conducted manually and was extremely time consuming, therefore only a two-minute section was reconstructed by 17. This particular window was chosen because the crowd density remains relatively stable throughout ^17^. A total of 274 individuals are observed during the window. For these individuals, all information required by the model is available: the time when each pedestrian entered in the station, the place of entry, the speed during each trajectory, and their final destination. Although these data are a much closer representation of true pedestrian dynamics than the hypothetical data used in similar work—e.g. 11, 14—it is important to note that they are somewhat dependent on the 2-minute window chosen by 17. Were another window chosen, for example one where the density changes dramatically, then the model might be less able to simulate the underlying dynamics. Future work should extend the research to more heterogeneous crowd data. All data used in this paper, including the video, the raw data and the processed data are publicly available ^17, 24^.

Figure 1 (a) illustrates several pedestrian trajectories obtained from these data after processing. Note that the southern portion of the station is not visible to the camera that was used to record the crowd, so only the visible part is simulated. Figure 1 (b) shows a representation of the model environment with 53 m width and 50 m height, 11 gates, and a 4 m radius information booth in the centre of the concourse. Some pseudo-real gates (gates from 7 to 10) are created to control the agent entry and exit regions when the agents cross the line that separates the visible region from the non-visible region. The average width of the ‘real’ gates (gates 0 to 6) is 13 *±* 5 m, so we create four 13.2 m gates to represent the southern boundary (gates 7 to 10).

Previous work on integrating DA and ABM have tended to analyse *macro* rather than *micro* patterns; i.e. the simulated data and observations are aggregated before being compared. There are advantages to this approach, such improved predictive ability, but here we choose to use the micro patterns (individual agent and pedestrian trajectories) because the use of macro patterns may mask underlying problems with our model or filter. For example, it would be possible for a model to simulate pedestrian density adequately but at the same time fail to simulate the real trajectories (the ‘wrong’ model happened to produce the ‘right’ result). The use of micro patterns provides a more accurate measure of the difference between the simulation and the real system, which is important for identifying the compatibility of DA and ABMs. Many of the surprising results we outline in later sections may have been masked if we had ignored the micro data.

We ultimately work towards the creation of tools that can be used to make predictions for real environments in real time. There are many instances where visitors who enter an environment are tracked or counted—for example as a passenger passes through a ticket gate to enter a public transport station—but fewer examples of tracking people while they actually move through the environment. This level of tracking also raises ethical and privacy questions. Therefore we do not provide the PF will all the information that is available in the GCT data and only provide the place of entry ( *entrance gate*) and the time that an individual entered ( *activation time*). The PF is not provided with the speed at which they will cross the environment ( *desired speed*) nor their final destination ( *exit gate*). So, when a pedestrian enters the environment, an agent is created with an explicit *entrance gate* and *activation time*, but the *desired speed* will be randomly selected from a truncated Gaussian distribution with mean of 1.6 m/s, a standard deviation of 0.6 m/s, and a minimum value of 0.05 m/s, and the *exit gate*, *g*, will be randomly selected with the following probabilities:


$$P(g|g_{in})=\{\begin{array}{c} \text{0}\\ \frac{1}{G-G_{in}} \end{array}\;\begin{array}{c} \text{if}\;g\;\text{and}\;g_{in}\;\text{are}\;\text{on}\;\text{the}\;\text{same}\;\text{side}\;\text{of}\;\text{the}\;\text{concourse,}\\ \;\text{if}\;g\;\text{and}\;g_{in}\;\text{are}\;\text{on}\;\text{different}\;\text{sides}\;\text{of}\;\text{the}\;\text{concourse,} \end{array}\;,\;(4)$$

where *g _in_* is the entrance gate, *G* = 11 is the total number of gates, *G _in_* is the total number of gates on the side of *g _in_*. These probability distributions describe the behaviour of real pedestrians observed in the GCT CCTV camera ^17^.

### PF

DA has a long history in the environmental sciences as a means of updating models with the most recent observations; see 26 for a review. Although it typically requires larger ensemble sizes than other methods ^14, 26, 27^, the PF is chosen here because it is non-parametric and hence better suited to systems that exhibit non-linear and non-Gaussian behaviour ^28^, such as agent-based models ^11^.

**Figure 2.**
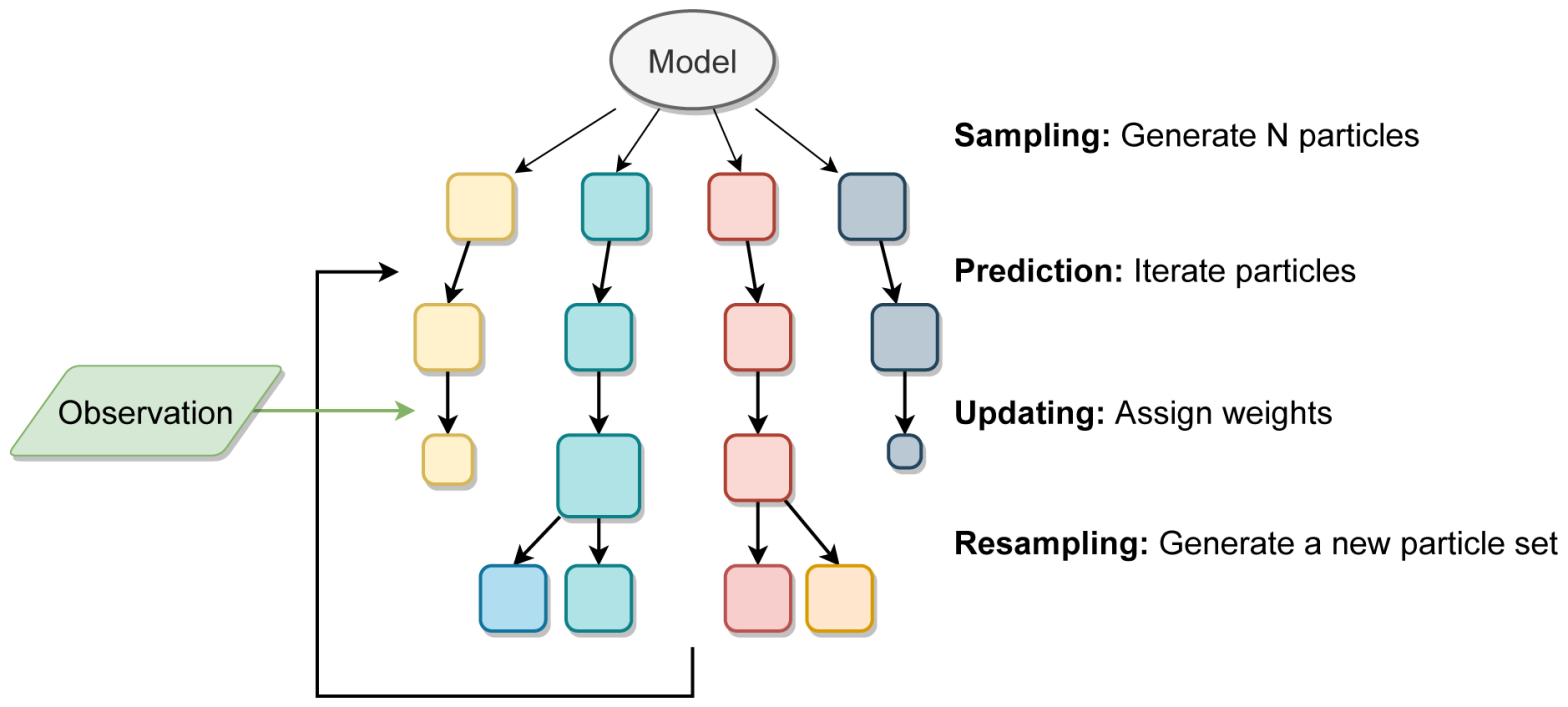
Illustration of a particle filter method. Each square represents a particle (full model realisation) and the size of the square represents the particle’s weight after comparison to the real data.

The goal of DA is to estimate a posterior distribution, and the PF does this in a brute force manner by creating an ensemble of *N* model instances, called ‘particles’, and then simultaneously running the particles for a number of iterations, termed the DA ‘window’ ( *prediction step*). When new observations arrive from the real system, the particles are paused, and the *updating step* is performed. For each particle, an importance weight is calculated that estimates how closely the particle corresponds to the given observations. Here, this weight is calculated for each particle by comparing the positions of the particle’s agents to those of the real individuals in the observed data. Finally, a resampling criteria based on particle weight is used to remove the worst performing particles—those that are least likely to have generated the observations—from subsequent iterations, whereas better performing particles are duplicated. These resampled particles represent the posterior distribution. Figure 2 shows an illustration of the PF process.

Each particle is a full realisation of a model with *n* agents each, and the particle *j* state at time *t* is defined as


$$\;S_{t}^{j}=\left\{s_{t}^{i}\;:\;i\in \{1,\ldots ,n\}\right\},\;(5)$$

where $s_{t}^{i}$ is the agent *i* state at time *t*. In this case, each particle is an instance of StationSim, and the agent’s state is defined by Equation 3.

Several resampling criteria can be used ^29^ to replace the worst performing particles with high-weight particles. Here, a variant called Sequential Importance Resampling (SIR) is used because it ranks higher in resampling quality and computational simplicity compared to other approaches ^30, 31^. The SIR PF approximate the filtering problem by an ensemble of weighted particles. Formally, a SIR PF at time *t* is


$$\;P_{t}=\left\{\left(S_{t}^{j},w_{t}^{j})\;:\;j\in \{1,\ldots ,N\right\}\right\},\;(6)$$

where $w_{t}^{j}$ is the corresponding weight – determined through the “variance of the weights”, i.e. weight is inversely proportional to squared distance between the prediction and the observation ^32^ – associated with particle *j* at time *t* and $\sum_{j=1}^{N}w_{t}^{j}=1$ .

There are two particularly well-known difficulties that PFs must overcome. Particle *collapse* occurs when one or a few particles have weights that are much higher than the others, so that the set of ‘useful’ particles is effectively much smaller than the total number of particles in the ensemble. With respect to particles that represent agent-based models, collapse may occur when the agents in most particles are not able to describe the observed behaviour in the underlying system, but a few particles have a large number of “good” agents (i.e. those that representing some of the observed pedestrians well). In this case, the few well-performing particles will have much higher weights than the others. The second difficulty, which often occurs after collapse, is particle *degeneracy*. A filter will degenerate if a few particles with high weights are resampled so often that the population of particles becomes near-identical. Many studies have found that the number of particles required to prevent particle degeneracy grows exponentially with the dimensionality of the model ^14, 26, 27^. This is particularly relevant for agent-based modelling, because as each agent typically has many distinct parameters, agent-based models usually have extremely high dimensionality. Although SIR helps to increase the spread of particle weights, it is often insufficient on its own ^26^. Here a process of random noise injection known as ‘jittering’ ^33^ is used to try prevent the collapse: at the beginning of each DA window a small amount of Gaussian noise is added to the ( *x*, *y*) positions of the agents.

***Adapted SIR PF.*** Unlike models in the natural sciences (where DA methods are normally applied) which are usually based on equations, ABMs are based on decision rules, and it is common for agents’ behaviour to be related to categorical parameters. As discussed in the Introduction, the later results will show that the vanilla PF implementation can actually *increase* the error in simulated agent positions after DA. This problem stems from the presence of categorical parameters in the model state vector; we will show that particles with the ‘correct’ agent destinations can be sampled out of the population early in the process, leaving no reliable particles in later stages of the simulation. The adapted PF involves a change to the resampling step that allows the worst performing particles to keep their inferred parameters ( *exit gate*, and *speed*), while replacing the position $\vec{r}$ of each agent with those of better performing particles. In other words, the positions of the agents in a particle are replaced, but their other parameters are not. This is a hybrid approach and aims to consider the two sources of uncertainty that are characteristic of an agent-based model: the random characteristics of the heterogeneous agents and the random choices that the agents make during the simulation. Formally, in the adapted SIR PF experiments, the state of agent *i* at time *t* is


$$\;s_{t}^{i}=\{{\vec{r}}_{t}^{i}\}.\;(7)$$

### Error metric

We use *mean distance* (MD) to quantify the prediction accuracy, defined as the average distance between agent positions predicted by the model and the positions of the pedestrian in the data:


$$\;\text{MD}=\frac{1}{n}\sum_{i=1}^{n}\sqrt{{\left(x_{Pi}-x_{Ai}\right)}^{2}+{\left(y_{Pi}-y_{Ai}\right)}^{2}},\;(8)$$

where *n* = 274 is the number of agents/pedestrians, *x _Pi_* and *y _Pi_* are the horizontal and vertical position of pedestrian *i* in the data, and *x _Ai_* and *y _Ai_* are the horizontal and vertical position of the agent *i* in the simulation. The mean distance between the predict and observed position is also used to determine the particle weights present in Equation 6. The precision is measured by the standard deviation (SD) defined as:


$$\;\text{SD}=\sqrt{\frac{1}{n}\;\sum_{i=1}^{n}{\left(\sqrt{{\left(x_{Pi}-x_{Ai}\right)}^{2}+{\left(y_{Pi}-y_{Ai}\right)}^{2}}-MD\right)}^{2}}.\;(9)$$

The results are presented in two different time references. The observation time reference gives the mean distance result in every frame. In each frame there are pedestrians at the beginning, in the middle or at the end of the trajectory, so this time reference gives an overview of the model prediction capacity. To identify at which points in the trajectory of each pedestrian the model prediction is better or worse, the result is also shown in the pedestrians’ time reference, where zero time is the time each pedestrian enters the scene.

### Experiment details

In total, four experiments were carried out; see Table 1. First, to evaluate the performance of the model two experiments were conducted without DA. In Experiment 1, all parameters were obtained from the data (unrealistic scenario as these parameters cannot be known in reality at real-time), then the same experiment was performed under a realistic scenario (only the time and entry gate for each agent are known). Then, two experiments were conducted with DA and under a realistic scenario.

**Table 1.** Experiments conducted using the StationSim model. Illustrates whether all parameters are given to the model (unrealistic scenario) or just *activation time* and *entry gate* (realistic scenario). Also shows which simulations include data assimilation and specifies the adopted method.

Experiment ID	Description	Data assimilation
Experiment 1	Unrealistic scenario (all parameters known, only the agents’ ( *x, y*) positions are uncertain)	None
Experiment 2	Realistic scenario ( *destination* and *desired speed* unknown)	None
Experiment 3	Realistic scenario ( *destination* and *desired speed* unknown)	SIR PF
Experiment 4	Realistic scenario ( *destination* and *desired speed* unknown)	Adapted PF

In all experiments 274 agents with radius of 0.5 m were simulated; 1 simulation step is equivalent to 1 frame from the CCTV camera (25 frames = 1 s). The information booth in the centre of the environment was modelled by a static disc with 4 m radius, and the collision time between an agent and the information boot was determined using Equation 1. In experiments 1 and 2, 5000 model samples were used. In experiments 3 and 4, 5000 particles were used, and DA—i.e. particle reweighting and resampling—was conducted every 100 simulation steps (4 s).

## Results and analysis

### Experiment 1: model evaluation

**Figure 3.**
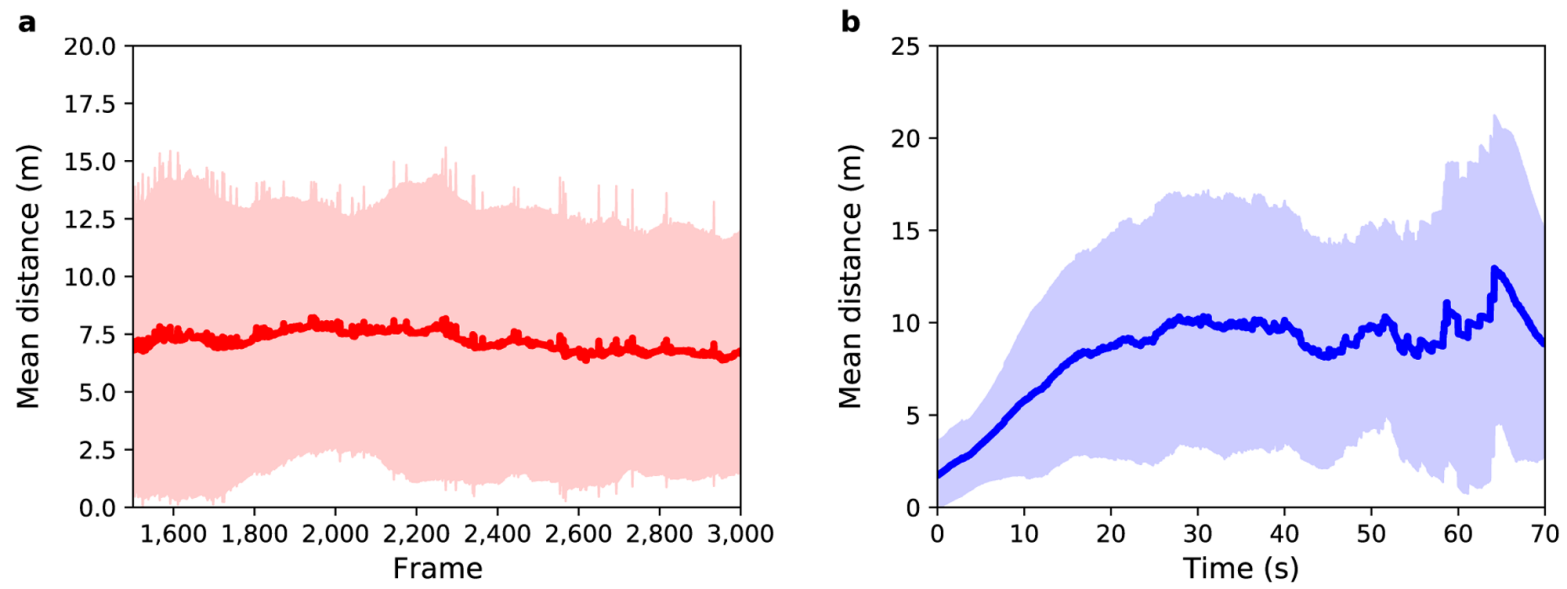
Experiment 1: ( **a**) Mean distance between agents and pedestrians (red solid line) as a function of sensor time. The light red region shows one standard deviation from the mean. ( **b**) Mean distance between agents and pedestrians (blue solid line) as a function of pedestrian time. The light blue region shows one standard deviation from the mean.

We first implement StationSim without DA in an unrealistic scenario to purely evaluate the performance of the model when all parameters ( *activation time*, *speed*, *entrance gate* and *exit gate*) are known. Figure 3 illustrates the average results over *N* = 5000 independent models.

Figure 3(a) shows mean distance as a function of sensor time. The average value (MD and SD) for the distance between agents and pedestrians over all selected frames is 7 *±* 6 m, i.e. about 14 times greater than a side-step that the agents take to avoid a collision. Therefore, the mean distance in this experiment cannot be explained solely by the random choices that agents make when crossing the station. Some pedestrians exhibit more complex trajectories than can be predicted by the model. On the other hand, as the standard deviation is almost equal to the mean difference, it is possible to infer that *some* pedestrians do have trajectories that are similar to those simulated in the model.

**Figure 4.**
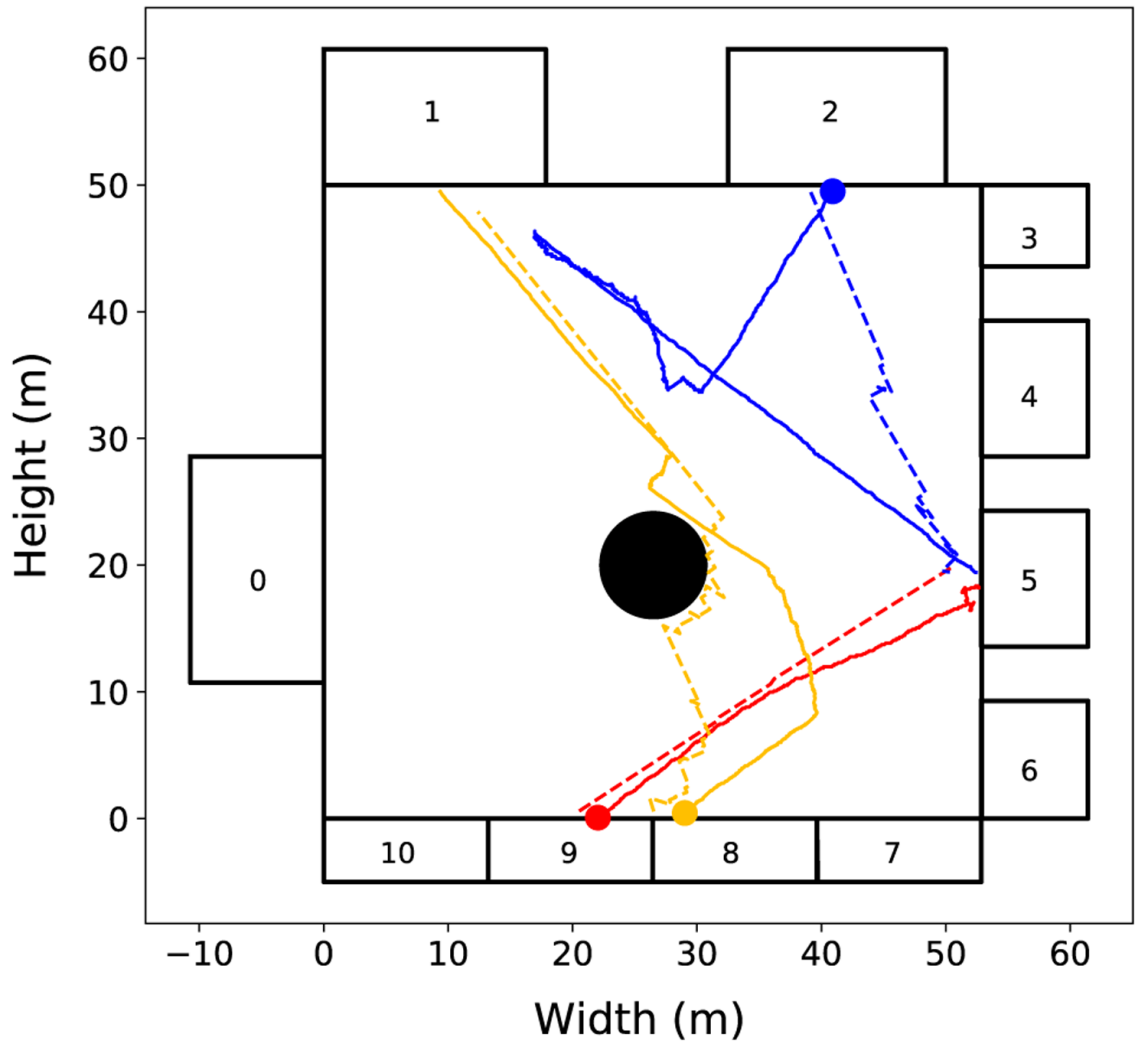
Experiment 1: Trajectories of three pedestrians (solid lines) and their respective agents (dashed lines). The circles mark the beginning of each pedestrian trajectory. Three cases of pedestrian movement are evident: (i) the red line illustrates a linear trajectory; (ii) the orange line illustrates a near linear trajectory with some obstacle avoidance; (iii) the blue line illustrate complex, non-linear trajectory.

To illustrate these cases, Figure 4 shows the trajectories of three pedestrians and their respective agents. The first scenario is illustrated by the pair of red lines. In this case, the pedestrian (solid line) performs an almost linear trajectory, as well as the movement of the agent (dashed line) predicted by the model. The second scenario is illustrated by the pair of orange lines. In this case, the pedestrian performs a more complex trajectory, but the change of movement direction is mainly related to interactions with other pedestrians or with elements of the environment. Because agents in the StationSim model can vary the direction of movement based on such interactions, even if a pedestrian executes a non-linear trajectory, it is possible for the agent to simulate this behaviour if the agent’s random decisions reflect real pedestrian behaviour. These two scenarios represent more than 70% of the observed trajectories ^17^. The third scenario (blue lines) represents the case where the pedestrian performs an even more complex trajectory that cannot be explained only by interactions with individuals or with the environment. In this case the agent is not able to adequately describe the pedestrian’s behaviour.

Figure 3(b) shows the mean distance as a function of pedestrian time, i.e. time 0 represents the point that the agent enters the system regardless of the frame that they actually enter in. For all agents the difference between their position and the position of their respective pedestrian is almost zero in the first few seconds of trajectory, but after 10 seconds the mean distance is 6 *±* 4 m. Such a difference in this small time interval is related to the complex trajectory that some pedestrians perform and that can not be predicted by the model. The mean distance increases with time until it reaches a value around 10 *±* 7 m after 31 s, which is mainly related to the width of the gates that have a mean value of 13 *±* 4 m — i.e. although the agents do not leave the station in the same position as their respective pedestrians, they leave through the same gate. This difference at the end of the trajectory can be related to the random choices that each agent makes during the simulation.

### Experiment 2: baseline scenario

**Figure 5.**
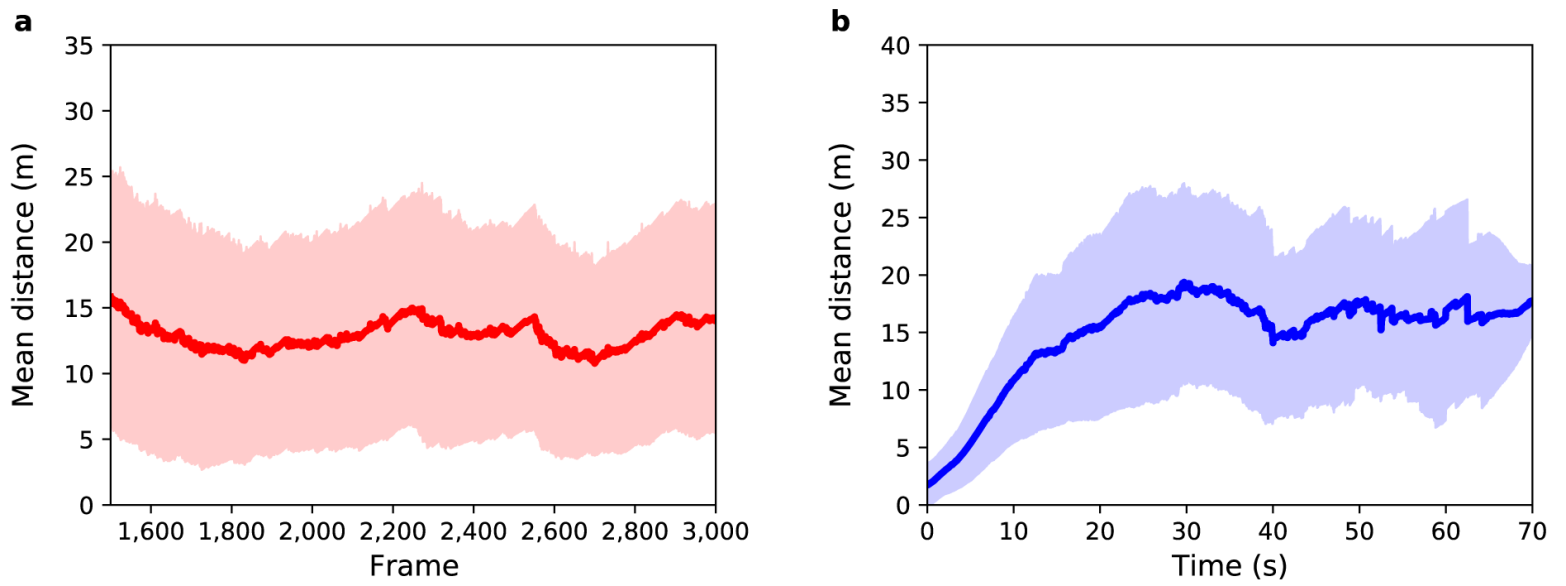
Experiment 2: ( **a**) Mean distance between agents and pedestrians (red solid line) as a function of sensor time. ( **b**) Mean distance between agents and pedestrians (blue solid line) as a function of pedestrian time. Shaded regions show one standard deviation from the mean.

As with the first experiment, Experiment 2 is also without DA, but now the *speed* and *exit gate* parameters are not provided to the model. Therefore, each time a model spawns an agent the values for these parameters are drawn from random distributions that were parameterised through analysis of the trajectory data. This is a baseline scenario to compare with future scenarios when DA is applied. Figure 5 shows the mean distance between agents and pedestrians for this experiment over *N* = 5000 independent models.

As with Experiment 1, Figures 5(a) and 5(b) show the mean distance as a function of sensor time and agent time respectively. With respect to Figure 5(a), the average value for the distance between agents and pedestrians over all selected frames is 13 *±* 8 m. In Experiment 1 it was 7 *±* 6 m. As expected, by randomly selecting the speed and exit gate parameters, the mean uncertainty in the prediction of pedestrian positions increases. The entrance gate and activation time were extracted from the data so the error at the beginning of the path of all pedestrians is small, but as the destination gate and speed were not known the trajectories diverge as the simulation runs. After 29 s, the mean distance reaches a value around 17 *±* 9 m, which is greater than the average width of the gates, indicating that the model is not able to correctly predict the exit gate for all agents.

### Experiment 3: SIR PF

**Figure 6.**
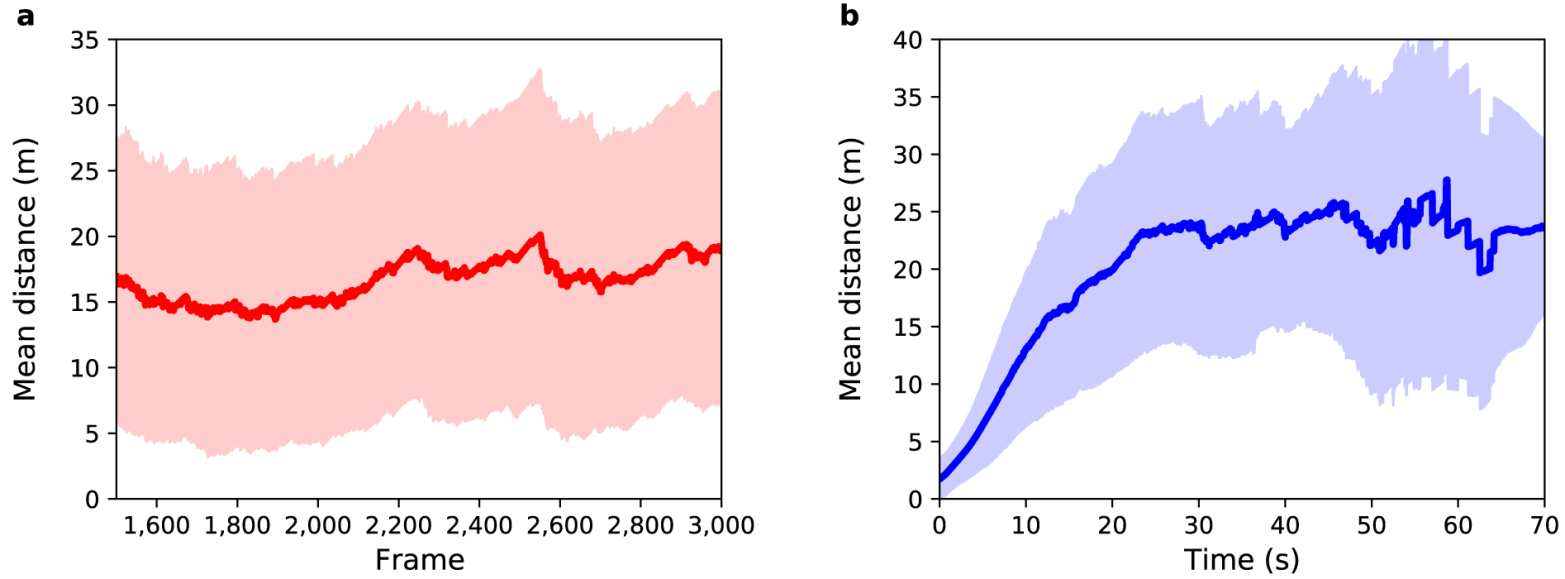
Experiment 3: ( **a**) Mean distance between agents and pedestrians (red solid line) as a function of sensor time. ( **b**) Mean distance between agents and pedestrians (blue solid line) as a function of pedestrian time. Shaded regions show one standard deviation from the mean.

Experiment 3 tests the use of the SIR PF as a means of reducing the difference between the real pedestrian traces and those simulated in StationSim. In this experiment *activation time* and *entrance gate* are known, but the *speed* and *exit gate* parameters are not provided to the particles. For this experiment 5000 particles are created with the same *entrance gate* and *activation time* as their respective pedestrian, while *speed* and *exit gate* parameters are randomly drawn. Figure 6 illustrates the result for this experiment.

Figures 6(a) and (b) show the mean distance as a function of sensor time and agent time respectively. The average value for the distance between agents and pedestrians over all frames shown in Figure 6(a) is 17 *±* 11 m. This value is greater than the observed in Experiment 2, where the average value was 13 *±* 8 m. Unexpectedly, in this experiment, DA using a PF *decreased* the accuracy and precision of the result! Figure 6(b) shows that during the first 10 s, the mean distance increases at a similar rate to that of Experiment 2. However, after 10 s the mean difference further increases until it reaches a value around 24 *±* 11 m. Although this result was not expected, it brings an important contribution to the understanding of the behavior of the PF when applied in ABMs.

In situations where there are few collisions, each agent in the StationSim model walks almost in a straight line towards their exit gate. However, as illustrated in Experiment 1, the real movement of pedestrians can be more complex. To understand a complex trajectory in terms of the movement allowed by the model, we can think of the pedestrian trajectory as the combination of several small almost linear trajectories. It would appear that the pedestrian walks almost in a straight line towards a gate, but after a short time they start walking towards a different gate. The more complex the trajectory, the more changes in the destination the pedestrian makes. In this way, the exit gate that generates the better trajectory at the *beginning* of the pedestrian’s movement is not the same exit gate at the *end* of the trajectory. The PF selected particles that better described the movement of pedestrians at the beginning of their trajectory. Then as the system evolves and it becomes clear that the gate chosen initially is not the one that the pedestrian is in fact heading to, all particles that might have included the ‘correct’ exit gate will have already been sampled out in the first moments of the experiment. Hence the movement of the agents begins to diverge quickly in relation to pedestrians’ movement. This phenomenon is related to particle degeneracy and is the main reason for the high error observed in Figure 6.

### Experiment 4: adapted PF

The result from Experiment 3 was unexpected and highlights a critical issue that arises when a PF is applied to an agent-based model and confronted with data from real human behaviour. Before concluding, we apply an adapted PF method to further investigate the problem highlighted in Experiment 3; namely that the particles with the ‘correct’ destinations can be resampled out of the population early in the process as the pedestrians do not follow straight paths. Recall that in the adapted PF, instead of completely removing the worst performing particles, agents in those particles will keep their inferred parameters ( *exit gate* and *speed*), while the dynamic ( *x*, *y*) position will be removed from subsequent iterations and replaced by the dynamic ( *x*, *y*) position from better performing particles.

**Figure 7.**
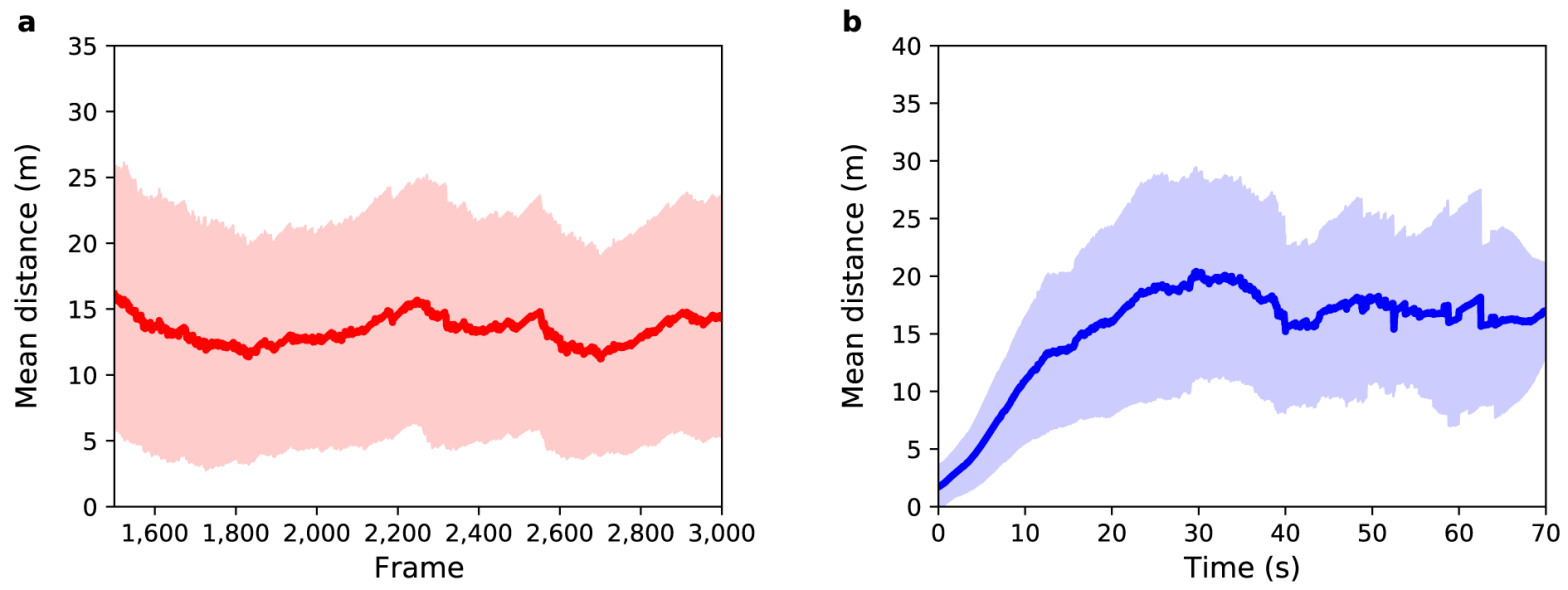
Experiment 4: ( **a**) Mean distance between agents and pedestrians (red solid line) as a function of sensor time. The light red region shows one standard deviation from the mean. ( **b**) Mean distance between agents and pedestrians (blue solid line) as a function of pedestrian time. The light blue region shows one standard deviation from the mean.

As with experiments 2 and 3, in Experiment 4 *activation time* and *entrance gate* are known, but the *speed* and *exit gate* parameters are drawn from random distributions that were parameterised through analysis of the trajectory data. Figure 7 shows the mean distance as a function of sensor time. The average value for the distance between agents and pedestrians over all frames is 13 *±* 9 m. This result shows a great reduction in uncertainty in relation to Experiment 3: the accuracy obtained was 23.5% (4 metres) better than that observed in Experiment 3, and the precision obtained was 18.2% (2 metres) better than that observed in Experiment 3. An overall improvement around 21%.

Although this result is exciting when compared to the result of Experiment 3, it is comparable to the baseline scenario (Experiment 2). To further improve the efficiency of the PF when applied to an ABM, some additional adaptations are necessary. This result is, however, enlightening when it comes to identifying the bottleneck of the combination of PF, ABM, and real pedestrian data: *the estimation of categorical parameters*. Future work should consider the possibility of changing categorical parameters with a certain probability.

## Discussion and conclusions

This paper extends the state-of-the-art by demonstrating how a PF can be used to incorporate real data in an agent-based simulation of pedestrians as they traverse the concourse of Grand Central Terminal in New York City (US). Although this is not the first work to use the combination of a PF and an ABM, it makes a valuable innovation through the use of real data. Up to now all attempts to unite DA and agent-based modelling have used hypothetical, ‘pseudo truth’ data ^11– 17^. This is important because we find that the complexity in the movements of real pedestrians seriously impact on the ability of the filter to optimise the model. This would not have been uncovered using toy data and is particularly important for agent-based models, as behavioural simplifications mean that virtual agents may not reflect the actions of real individuals. When using the traditional SIR PF, the filter selected particles that better described the movement of pedestrians at the beginning of their trajectory, but when pedestrians changed their direction of movement there were no longer any particles capable of describing this new direction. By understanding the behaviour of the PF when applied to an agent-based model, we were able to apply a method capable of reducing uncertainty by about 21% in relation to the uncertainty observed with the SIR PF. Despite being an expressive result, more precise methods still need to be developed as this improvement is marginal when compared to the baseline experiment.

In the Methods section we explain that particle degeneration and collapse can occur when the spread of the population of particles becomes very narrow—i.e. all particles in the population become very similar. It is well documented that the particle ensemble size needs to maintain a constant error to prevent collapse and it scales exponentially with the dimensions of the state space ^27^. The agent-based model here, like many agent-based models, has high dimensionality: 274 agents each with two unknown parameters ( *exit gate* and *speed*) and a dynamic ( $\vec{r}$ = *x*, *y*) position that becomes uncertain when agents collide. Although we added Gaussian noise at the beginning of each DA widow to all the agents’ positions within a particle to provide some resilience to the unexpected non-linear pedestrian movement and prevent filter collapse and state degeneracy, some particle collapse was observed. More specifically, the results point to the need for methods that add a fluctuation to the categorical parameters, as well as the use of methods that support the high dimensionality of ABMs.

More advanced methods do exist that try to circumvent this issue, one of which is local particle filtering (LPF). LPF takes the approach of splitting the state space into sub-states ^34^. In this case the weighting and resampling steps are performed locally within a region and the full state of the system is defined by the tensor product of all sub-states. In the case of ABMs for pedestrian movement it is difficult see how to use LPFs without changing the overarching output from predictions of individual pedestrian movements to a wider output of predicting crowd movement and aggregated characteristics which may be better approximated by aggregate models rather than ABMs in the first place. Another potentially useful method is the Particle Flow Filter (PFF). In the PFF, particles iteratively transition from the prior to the posterior state (without the resample process), consequently avoiding the degeneracy problem. In addition to being promising for high-dimensional systems, the PFF developed by Pulido *et al.* ^35, 36^ is particularly promising, since it is compatible with nonlinear observations. Future work will experiment with this filter.

One hyper-parameter that can be further experimented with is the length of the DA window. This represents the time that passes between the PF receiving observations and resampling its particles. Here the DA window was 4 seconds (represented by 100 model iterations). In theory, shorter windows will allow less time for the particles to diverge from the real system, so should lead to lower uncertainty in the filtering, but, as shown in Experiment 3, as soon as the particles exhibit low variability in categorical parameters, the result will quickly diverge from reality. Therefore, using a shorter DA window should further highlight the difficulty of the filter handling ABMs’ categorical parameters. Besides that, it may not be possible to implement shorter DA windows in the real world where there may be technical constraints on the rate that observations can be physically collected and delivered to the PF. Although larger ensemble sizes should be able to mitigate some of the increased uncertainty that would be introduced with larger assimilation windows, if the window were too large then pedestrians might be able to enter and leave the system without being observed at all. In these circumstances it would be extremely difficult to implement a real-time simulation of the system.

In terms of potential future applications, one of the most interesting avenues to explore could be the use of pedestrian ABM coupled with a DA algorithm to predict crowd behaviour in emergency situations. If the model and DA algorithm are working well, then we should be able to make reliable short-term predictions under ‘normal’ conditions. This would allow the identification of emerging issues before they become problematic, such as over-crowding in certain parts of the environment. These emerging problems could, of course, be managed directly, but there is also an opportunity to allow a model to try to resolve them automatically. However, a model that has been designed for ‘normal’ conditions may break down when an emergency causes individuals to behave differently, so there may need to be an alternate simulation that is deployed to deal with emergency situations specifically.

In summary, this paper has experimented with the coupling of a DA algorithm (the PF) and an agent-based model of a pedestrian system. This is important because, as with many social systems, the underlying complexity and uncertainty inherent in crowds means that model outputs will quickly diverge from reality, so static models will not be able to simulate the dynamics of a system in *real time*. We show that the standard SIR PF struggles to control for the complex behaviours of real pedestrians, but some relatively small adaptions to the basic method might make it more amenable. The results have important implications for those interested in creating real-time simulations to support the day-to-day management of places that are characterised by high volumes of pedestrians.

## Data availability

### Source data

The pedestrian raw data ^24^ and the further processed data ^17, 22^ that support the findings of this study are publicly available.

## Software availability

Source code available from: https://github.com/patricia-ternes/dust/tree/v0.2

Archived source code at time of publication: https://doi.org/10.5281/zenodo.5556093 ^37^

License: MIT License

The source code to run the StationSim model and the PF experiments can be found in the main ‘Data Assimilation for Agent-Based Modelling’ (DUST) project GitHub repository ^22^. Specifically, codes, scripts and instructions to run the experiments are available in: https://github.com/Urban-Analytics/dust/tree/main/Projects/ABM_DA/ experiments/gcs_experiments.
