# Supplementary material for: Data assimilation and agent-based modelling: towards the incorporation of categorical agent parameters
Source: Open Res Eur. 2022 Jul 20;1:131. Originally published 2021 Oct 27. [Version 2] doi: 10.12688/openreseurope.14144.2 (PMC10445938; doi:10.12688/openreseurope.14144.2)
Supplement: Peer review history

# Peer review: Reviewer response for version 2

**DOI:** 10.21956/openreseurope.16172.r29734

**Published:** 2022-08-01

**Authors:** Manuel Pulido

**Affiliations:** 1 Department of Physics, Universidad Nacional del Nordeste, Corrientes, Argentina

My main concerns have been addressed in this new version of the manuscript, so that to me it could be accepted for indexing.

If applicable, is the statistical analysis and its interpretation appropriate?

Partly

Is the study design appropriate and is the work technically sound?

Yes

Is the work clearly and accurately presented and does it engage with the current literature?

Yes

Are the conclusions drawn adequately supported by the results?

Yes

Are sufficient details of methods and analysis provided to allow replication by others?

Yes

Are all the source data and materials underlying the results available?

Yes

Reviewer Expertise:

NA

I confirm that I have read this submission and believe that I have an appropriate level of expertise to confirm that it is of an acceptable scientific standard.

# Peer review: Reviewer response for version 1

**DOI:** 10.21956/openreseurope.15249.r28468

**Published:** 2022-02-17

**Authors:** Manuel Pulido

**Affiliations:** 1 Department of Physics, Universidad Nacional del Nordeste, Corrientes, Argentina

This manuscript develops a data assimilation system that is composed of a classical particle filter and an agent-based model. The agent-based model describes pedestrians walking around the concourse of a terminal. The technique is validated using real data from a terminal in New York City. ABMs are very descriptive models at the expense of having multiple free parameters that require to be properly set for realistic simulations. Therefore there is a current need for the development of callibration techniques that may constrain parameters and the state of the system close to the observations. The present manuscript is a pertinent effort along this line. One challenge that the authors had to deal with in their work is the presence of categorical variables (destination gate of the pedestrians) that are difficult to be statistically represented in data assimilation. They propose a relaxation of the resampling step. The manuscript is well written and the proposed technique and evaluation experiments are relevant so I recommend the indexing of the article with some comments that the authors may consider for the revised versions.

 The authors have previous work on the use of the ensemble Kalman filter for data assimilation in ABMs. The motivation on why they chose a particle filter in this work instead of other Gaussian techniques (e.g. ensemble Kalman filter) requires some further discussion. In the article, they mention nonlinearities as a motivation. Are the nonlinearities of the model statistically important? How do the authors evaluate this point? My (probably wrong) intuition was that because the system was highly unpredictable since pedestrian trajectories have multiple motivations (many not influenced by the modeled variables), because of that data assimilation schemes that may correct the predicted trajectories of the individuals and exit gate using observations could be more suitable. At the same time, particle filters are promising because the only coupling between observations and predictions is via the observational likelihood so that state evolution is enterely based on the simulations. In this sense, particle filters look a promising venue for epidemiological ABMs in which state corrections in the filter are difficult (Cocucci *et al *2022).

 Perhaps some credit in the introduction to the original works on particle filters (e.g. Gordon *et al* 1993 etc) should be given in the manuscript. The interest in the particle filter in geoscience applications is much more recent (and I would say it is still an open field for those high-dimensional applications).

 What is the observation likelihood that the authors are using in the particle filter? Are observations assumed to be without error? The weighting factor is the inverse of the mean distance. Is this more suitable than a Gaussian/Mahalonobis distance?.

 As far as I can see the impact of observations on the exit gate attribute is rather indirect: the trajectory likelihood. I wonder if one could incorporate the "mean" direction information as a proxy for the exit gate (this could be also an observable variable).

 Standard parameters in particle filters are known to produce sample degeneracy. To avoid this problem an artificial random evolution is added to the parameters so that they can explore the parameter space (Liu and West 2001). I wonder if some kind of stochasticity may be added to the exit gate (particularly at the initial times) to avoid the problem of the particle filter related to particle degeneracy.

 I was expecting some further analysis of the assimilation experiments (3 and 4). Does the performance of the filter depend on the particle number? Could you show the number of effective particles and compare its evolution between Exp 3 and 4.

 About the conclusions, my impression is that particle flow filters (e.g. Pulido and vanLeewen 2019) may be potentially more interesting to apply than local particle filters. Localization does not appear to be suitable for this application, I think it would be very hard to determine which agent states are independent and which are correlated. On the other hand, the goal of particle flow filters is to avoid particle impoverishment moving the particles toward the posterior density.

 Further general comments/discussion:

 It seems that there are no learnable parameters in the filter apart from exit gate and speed? Could the authors foresee some further parameters that are correlated to observations to be incorporated in the state space? Another related point for further discussion in the manuscript. Will the system be able to predict any urgency situation that alters the standard pedestrian trajectories? This could be one of the most important features to model and predict.

 Is it essential real-time data assimilation in this application? Could the system be conditioned with near-future observations to improve the performance of the filter/smoother?

 References:

 Cocucci *et al*  2022: Inference in epidemiological agent-based models using ensemble-based data assimilation. arXiv:2111.00073 (in revision in Plos One).

 Gordon, N.J., Salmond, D.J. and Smith, A.F., 1993, April. Novel approach to nonlinear/non-Gaussian Bayesian state estimation. In IEE Proceedings F-radar and signal processing (Vol. 140, No. 2, pp. 107-113). IET.

 Liu, J. and West, M., 2001. Combined parameter and state estimation in simulation-based filtering. In Sequential Monte Carlo methods in practice (pp. 197-223). Springer, New York, NY.

 Pulido, M. and van Leeuwen, P.J., 2019. Sequential Monte Carlo with kernel embedded mappings: The mapping particle filter. Journal of Computational Physics, 396, pp.400-415.

If applicable, is the statistical analysis and its interpretation appropriate?

Partly

Is the study design appropriate and is the work technically sound?

Yes

Is the work clearly and accurately presented and does it engage with the current literature?

Yes

Are the conclusions drawn adequately supported by the results?

Yes

Are sufficient details of methods and analysis provided to allow replication by others?

Yes

Are all the source data and materials underlying the results available?

Yes

Reviewer Expertise:

data assimilation

I confirm that I have read this submission and believe that I have an appropriate level of expertise to confirm that it is of an acceptable scientific standard, however I have significant reservations, as outlined above.

## References

[1] : Inference in epidemiological agent-based models using ensemble-based data assimilation (in revision). *Plos One* .2022; Reference source 10.1371/journal.pone.0264892PMC889671335245337

[2] : Novel approach to nonlinear/non-Gaussian Bayesian state estimation. *IEE Proceedings F Radar and Signal Processing* .1993;140(2) : 10.1049/ip-f-2.1993.0015 10.1049/ip-f-2.1993.0015

[3] : Combined Parameter and State Estimation in Simulation-Based Filtering.2001; 10.1007/978-1-4757-3437-9_10 197-223 10.1007/978-1-4757-3437-9_10

[4] : Sequential Monte Carlo with kernel embedded mappings: The mapping particle filter. *Journal of Computational Physics* .2019;396: 10.1016/j.jcp.2019.06.060 400-415 10.1016/j.jcp.2019.06.060

# Peer review: Reviewer response for version 1

**DOI:** 10.21956/openreseurope.15249.r28262

**Published:** 2022-01-31

**Authors:** Nikolai W. F. Bode

**Affiliations:** 1 Department of Engineering Mathematics, University of Bristol, Bristol, UK

This contribution is concerned with testing data assimilation techniques for calibrating an agent-based model on an openly available data set of pedestrian trajectories. Overall, this is an interesting contribution that is presented well and I could not find any obvious technical issues. My comments are mainly about the broader context and approach of this work.

 The data selection was explained only with reference to computation times. It would be useful to discuss limitations of this data selection in terms of how representative of the overall data set it is: e.g. what would the advantages/disadvantages of choosing two separate 1-minute sections instead of one 2-minute section have been?

 Some aspects for model parameterisation are surprising, given that availability of trajectory data is assumed. For example, the preferred speed could be informed by taking speed measurements (a realistic proposition with current technology). Similarly, the exit gate estimation could be informed by observed movement directions of agents. This more informed parameterisation may reduce the number of particles needed in PF. As it stands, PF is asked to achieve a lot. If I misunderstand why the approach outlined above is not what is intended here, then an explanation for this would be useful.

 In general, the approach outlined attempts more than just model calibration. However, model calibration is a useful comparison and it would therefore be useful to discuss some of the work that has attempted to calibrate models for pedestrian movements, as this is rather difficult and may help to contextualise this work.

 Predicting/matching the trajectories of individuals is very ambitious. As the authors show, there are all sorts of behaviours that most models cannot predict or do not even consider.

 Most approaches for DA will attempt to reduce the complexity of the problem (e.g. consider weather predictions on a grid). I think the authors should discuss to what extent this could be done for the data they consider. E.g. movement could be predicted on a coarser grid, only pedestrian densities could be predicted, etc.

 Such an approach may improve prediction success substantially, even if the same model is used.

 I haven't had the time to look at the code and can therefore not comment on it.

If applicable, is the statistical analysis and its interpretation appropriate?

Yes

Is the study design appropriate and is the work technically sound?

Yes

Is the work clearly and accurately presented and does it engage with the current literature?

Partly

Are the conclusions drawn adequately supported by the results?

Yes

Are sufficient details of methods and analysis provided to allow replication by others?

Yes

Are all the source data and materials underlying the results available?

Yes

Reviewer Expertise:

Agent-based models, model calibration, pedestrian dynamics

I confirm that I have read this submission and believe that I have an appropriate level of expertise to confirm that it is of an acceptable scientific standard.
